# Preparation and Application of a Multifunctional Interfacial Modifier for Ramie Fiber/Epoxy Resin Composites

**DOI:** 10.3390/polym15183800

**Published:** 2023-09-18

**Authors:** Liyue Zhang, Jingkai Liu, Jinyue Dai, Xufeng Zhang, Xiaoling Liu, Xiaoqing Liu, Xiaosu Yi

**Affiliations:** 1 New Material Institute, University of Nottingham Ningbo China, Ningbo 315100, China; zhangliyue@nimte.ac.cn (L.Z.); xiaoling.liu@nottingham.edu.cn (X.L.); 2 Key Laboratory of Marine Materials and Related Technologies, Ningbo Institute of Materials Technology and Engineering, Chinese Academy of Sciences, Ningbo 315201, China; 3 College of Materials, Beijing Institute of Technology, Beijing 100081, China

**Keywords:** ramie fiber, epoxy resin composites, mussel-inspired, interfacial properties, flame retardant

## Abstract

A multi-functional modifier, which could improve the mechanical and thermal performance simultaneously, is significant in composites production. Herein, inspired by the chemistry of mussel, an interfacial modifier named FPD was designed and synthesized through one simple step, which was attached by three functional groups (including catechol, N-H bond, and DOPO). Due to the innate properties of each functional group, FPD played multiple roles: adhere to the ramie fibers from catechol and cure with the epoxy resin from -NH-, an antiflaming property from DOPO, and the compatibilizer between ramie fibers and epoxy resin was also improved by changing the polarity of ramie fiber. All of the above functions can be proved by means of water contact angle (WCA), atomic force microscope (AFM), and scanning electron microscopy (SEM), etc. After solidification, the ramie fiber/epoxy composites demonstrated superior performances in terms of good mechanical properties and excellent flame retardant property. With the addition of 30 wt.% FPD, the tensile strength and modulus of the ramie/epoxy composite showed an improvement of 37.1% and 60.9%, and flexural strength and modulus of the composite were improved by 8.9% and 19.3% comparing with no addition composite. Moreover, the composite could achieve the goal for V-0 rating in the UL-94 test and LOI value was 34.6% when the addition of FPD reached 30 wt.%. This work provided us with an efficient method for fabricating nature fiber/epoxy composites with good properties.

## 1. Introduction

Nowadays, under shortage of resources and aggravated environmental pollution, efficient use of renewable resources is constantly strengthened. Unlike glass fibers and carbon fibers, plant fibers as environmental reinforcements of composites have the unique advantages of being lightweight, low cost, biodegradable, and renewable [1]. Particularly, ramie fiber might satisfy the requirements of reinforced fiber because of its lower specific density (1.5 g/cm^3^) and competitive tensile modulus of 61.4–128 GPa, which is close to E-glass fiber (70 GPa) and higher than that of flax, jute, and sisal fibers (27.6, 13–26.5 and 9.4–22.0 GPa) [2,3,4,5]. In terms of polymer, epoxy resin represents the most widely utilized matrix due to its excellent dimensional stability, superior chemical resistance, and mechanical qualities [6]. Hence, ramie fiber reinforced epoxy resin composite is undoubtedly promising.

As we know, each of the composites possess benefits and drawbacks. On the one hand, the high flammability of both plant fiber and epoxy resin as well as the “wick effect” restrict their use to industries with strong fire safety rules [7,8]. To this end, flame-retardant compounds must be incorporated into composites by incorporating flame retardants (FRs) into polymer or fibers [9,10]. Up to now, 9,10-Dihydro-9-oxa-10-phosphaphenanthrene-10-oxide (DOPO) was shown to be one of the most efficient in terms of flame retardancy and gained growing attention both in academia and industries [11,12]. To avoid impairing the mechanical performance caused by the direct inclusion of flame retardant and consider the characteristic of epoxy resin, synthesizing a new compound containing DOPO and an imino group incorporated into epoxy resin as a curing agent is a potential solution [13,14,15]. On the other hand, the hydrophilic natural fibers could not be inherently compatible with hydrophobic epoxy resin, which would lead to weak interfacial adhesion between the fiber and the matrix [16] and further result in poor mechanical for composites [17,18,19,20]. In many applications, surface modification including alkali treatment, surface oxidation, chemical grafting, plasma irradiation, and ultraviolet grafting is widely employed to improve the adhesion [21,22] or mechanical interlocking [23]. However, most of the above treatments may sacrifice the fiber’s mechanical properties and structural integrity [24]. At present, silane coupling agents as a traditional and excellent damage-free surface modifier were proven in research [25]. However, all these methods were constrained by the fact that they could not improve mechanical and thermal properties simultaneously [17]. In this way, a multi-functional modifier, which could match the above requirement, is necessary. 

In recent years, a mussel-inspired coating strategy attracted much attention because of its ability of easy implementation and general universality [26,27,28], which some are convinced to be a potential way to modify matrix in interfacial functionalization [29,30]. Chen et al. [24] prepared polyethyleneimine/catechol [31]-modified PET fiber-reinforced composites by mussel inspiration, and the analysis revealed that the interfacial property was significantly improved compared to the untreated PET-reinforced composite. Unfortunately, it is still rare that research focuses on new compounds with mussel inspiration and multi-functional ability to produce fiber-reinforced composites. 

Based on the above analysis, the ideal modifier that may satisfy these requirements should contain at least three functional structures including catechol, N-H, and DOPO. Specifically, catechol can adhere the ramie fabric similarly to mussel and the N-H cure the epoxy resin during curing process, and the two special structures could form chemical links between the reinforcement and matrix to enhance the interfacial performance. In addition, DOPO can endow the compound flame-retardant characteristic. In fact, a chemical compound named DPDDM, which contains these three structure was reported before [14]. Nevertheless, it was utilized as a co-curing agent for epoxy resin and cannot dissolve in any organic solvent except DMF due to its strong rigidity, which indicates that it cannot achieve the adhesion function of catechol and be used for the modification of composite materials.

In this study, a novel compound soluble in low-boiling solvent (tetrahydrofuran) was designed and synthesized successfully. Moreover, the hydrophobicity of ramie fabric after being modified by FPD was improved, and the compatible was also promoted in the process of making the composite, furthering the interfacial properties of the obtained composites facilitated. Concurrently, the composite also displayed outstanding fire safety performance. This work offers an effective approach to fabricate strong interfacial and flame-retardant ramie-reinforced epoxy composites.

## 2. Materials and Methods

### 2.1. Materials and Chemicals Used

Ramie fabric was purchased from Hunan Huasheng Dongting hemp Industry Co., Ltd., the areal density is 135.1 g/m^2^, and 3,4-dihydroxybenzaldehyde, aniline, polyethyleneimine (PEI), and 4,4′-diaminodiphenylmethane (DDM) were obtained from Aladdin Reagent Co., Shanghai, China. The 9,9-Bis(4-aminophenyl)fluorene was purchased from Shenzhen Star Kaiyue Biotechnology CO., Ltd., Shenzhen, China. Diglycidyl ether of bisphenol A (trade name DER-331, abbreviated as DGEBA, viscosity less than 1 Pa·s) with epoxy value around 0.51–0.53 mol/100 g was purchased from Dow Chemical Company. The 9,10-dihydro-9-oxa-10-phosphaphenanthrene-10-oxide (DOPO, 98%) was bought from Guizhou Yuanyi Mining Group Co., Ltd., Guiyang, China. Tetrahydrofuran and ethyl alcohol was acquired from Sinopharm Chemical Reagent Co., Ltd., Shanghai, China. All the materials were used as received without any further purification. 

### 2.2. Synthesis of 4,4′-(9H-fluorene-9,9-diyl) Dianilineand 6,6′-((((9H-fluorene-9,9-diyl)bis(4,1-phenylene))bis(azanediyl))bis((3,4-dihydroxyphenyl)methylene)) Bis (dibenzo (c,e)(1,2)oxaphosphinine 6-oxide) (FPD)

**Figure 1:**
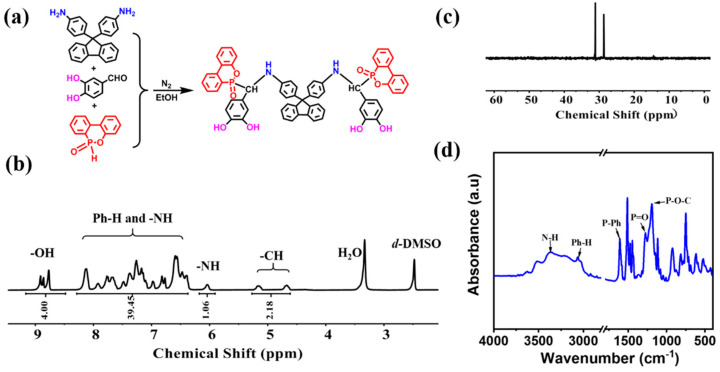
(**a**) Synthesis illustration and chemical structure of FPD; (**b**) ^1^H NMR, (**c**) ^31^P NMR, and (**d**) FT-IR spectra of FPD.

According to the synthetic procedure showed in Figure 1a, in a 250 mL three-necked flask equipped with a reflux condenser, a magnetic stirrer, and nitrogen atmosphere, 9,9-Bis(4-aminophenyl)fluorene (17.4 g, 0.05 mol), and 3, 4-dihydroxybenzaldehyde (13.8 g, 0.1 mol) were dissolved in 300 mL ethanol. The mixture was heated up to 80 °C and kept at this temperature for 12 h. After that, DOPO (21.6 g, 0.1 mol) was added and continued to react for 12 h at 80 °C; then, it was cooled to room temperature and the aqueous solution was added dropwise into hot water. The product was filtered and washed with hot water for three times. Then, it was dried at room temperature for 24 h under a vacuum and a light-yellow solid powder (FPD) was obtained (yield: ~85.7%). ^1^H NMR (DMSO-d6, ppm): δ = 9.2–8.5 (O-H), 8.3–6.4 (Ar-H and N-H), 5.2 (N-H), and 5.16 and 4.67 (C-H).

### 2.3. Fabrication of FPD on Ramie Fabric by Mussel Inspiration

According to the adhesion mechanism of dopamine, which is polymerized in an alkaline solution and deposited on the surfaces of various materials, for catechol, it would be polymerized and deposited with polyamine in an alkaline solution. In this way, ramie fabric was first immersed in an aqueous solution of 1 mg·mL^−1^ for 20 min to deposit a layer of PEI to provide -NH_2_ on the surface of ramie [32]. After being washed with deionized water, the PEI-coated fabric was squeezed to remove the adsorbed water and then dried in an oven for 4 h at 65 °C. Then, the FPD modification of the ramie fiber producer occurred in a THF solution containing various contents of FPD (10 wt.%, 20 wt.%, and 30 wt.% for the composites weight), adjusting the pH value to 8.5 with NaOH solution, and then immersed the fiber until the THF volatilized. After completion of the reaction, the FPD-grafted ramie fabric was washed with methyl alcohol and dried in an oven, the flame-retardant fabric was obtained and named RF-1, RF-2, and RF-3, respectively, and the pristine ramie fabric was named RF-0.

### 2.4. Preparation of Ramie-Epoxy Resin Composites

For fabrication of each composite, twelve layers of four kinds of ramie fabrics measuring 150 mm× 150 mm were cut and dried in a convection oven. The epoxy resin (DER-331) was mixed with the hardener (DDM) and the transparent mixture was obtained; then, the composite material was prepared by a vacuum-assisted resin transfer molding process. The curing reaction was conducted at 90 °C for 2 h, 110 °C for 2 h, and then 130 °C for 2 h. The laminates were obtained after natural cooling to room temperature. The resin content was kept at 55–60 wt.%. The composites that obtained FPD-treated ramie fiber with different loading are marked as RFEPC-0, RFEPC-1, and RFEPC-2 and RFEPC-3, respectively. The detailed formulas were shown in Table 1. Test specimens were prepared by cutting and machining the composites to the standard size for the characterization of tensile and flexural properties (strength and modulus), flame retardancy, etc.

### 2.5. Characterization

^1^H, ^13^C, and ^31^P nuclear magnetic resonance (NMR) spectra were collected on a 400 MHz Bruker AVANCE III spectrometer (Bruker, Switzerland) at 25 °C with DMSO-*d_6_* as solvent and tetramethyl silane as internal standard reagent. The attenuated total reflection-Fourier transform infrared (ATR-FTIR) spectrum was recorded on an infrared spectrometer (Cary660 + 620, Agilent, La Jolla, CA, USA) from 4000 to 400 cm^−1^ with a resolution of 4 cm^−1^ at room temperature. The X-ray diffraction spectrometry (XRD) was conducted to research the crystallization of samples by a Bruker D8 advance diffractometer with Cu Ka radiation (1.54 Å) in the range of 5°–60°. The static water contact angle (WCA) in the air was performed using a contact angle-measuring instrument (OCA25, Dataphysics, Filderstadt, German) with a 2 μL DI water droplet at room temperature. The results were taken from the average of the WCAs of five different parts of each sample. All the atomic force microscope (AFM, Bruker, Ettlingen, Germany) measurements were performed in PeakForce quantitative nanomechanical mapping (PF-QNM) mode at ambient conditions. The adhesive force measurements were performed with the spring constant (SNL-10, k = 0.35 N/m) of a standard silicon nitride AFM tip to quantitatively determine adhesion forces between the fibers and AFM tip according to the adhesion force–distance curves. Differential scanning calorimetric (DSC) measurements were conducted on a Mettler Toledo MET DSC (METTLER TOLEDO, Küsnacht, Switzerland) at heating rates of 5, 10, 15, and 20 °C/min from 25 to 250 °C. Thermogravimetric analyses (TGA) were performed using a Mettler Toledo TGA/DSC1 (METTLER TOLEDO, Switzerland) with a 20 °C/min heating rate from 25 to 800 °C under a nitrogen atmosphere (flow rate: 25 mL/min). The limiting oxygen index (LOI) values of ramie fabric composites were acquired by a digital oxygen index apparatus (Jiangning Analytical Instrument Co., Ltd., Nanjing, China) according to the ASTM D2863-2008 standard. The size is 150 mm (length) × 6.5 mm (width). A vertical flame test was conducted on an automatic vertical burning tester (Jiangning Analytical Instrument Co., Ltd., China). Vertical flame tests of composites were performed according to ASTM D3801 standard with sample dimensions of 130 × 13 × 3 mm. The heat release of the composites was analyzed with a cone calorimeter test (CCT) (Fire Testing Technology, East Grinstead, UK). Sample dimensions of 100 mm × 100 mm were exposed to a heat flux of 35 kW/m^2^ according ISO 5660-1. The surface morphologies of ramie samples before and after modification, fracture surfaces after tensile test, and char residues after CCT were observed using field-scanning electron microscopy (FE-SEM, EVO18, Zeiss, Jena, Germany) equipped with an energy-dispersive spectrometer. Thermogravimetric analysis–infrared spectrometry (TG-IR) analysis was carried out on a TGA 8000 thermogravimetric analyzer coupled with a two-Clarus SQ8T spectrometer (PerkinElmer, Valencia, CA, USA) from room temperature to 800 °C with a heating rate of 10 °C/min. The Raman spectra were collected from a Raman spectrometer via Reflex (Renishaw, Britain) to further characterize the char residues after CCT. The flexural and tensile properties were performed by universal material testing machine (5567, Instron, Shanghai, China) as per ASTM standard D-3039 method and ASTM D-2344, respectively. The impact strength was carried out on a CEAST 9050 impact resistance device (Instron, Norwood, MA, USA).

## 3. Results and Discussion

### 3.1. Synthesis of FPD and Characterization of Mussel-Inspired Ramie Fabric

The synthesis illustration and chemical structure of FPD were displayed in Figure 1a. To confirm the success synthesis of FPD, the ^1^H, ^13^C and ^31^P NMR, FT-IR spectra were collected. Figure 1b and Figure S1 showed the ^1^H NMR and ^13^C NMR spectra of FPD, where the characteristic signals were the powerful evidence for the successful synthesis. As shown in Figure 1b, the characteristic signals for -CH could be seen at 5.16 and 4.67 ppm, signals for Ph-H and -NH were at 8.3–6.4 and 6.05 ppm, and signals for -OH could also be observed at 9.2–8.5 ppm. To further confirm whether the -NH was in 8.3–6.4 ppm, the ^1^H NMR with D_2_O was conducted in Figure S2, the -OH 9.2–8.5 ppm and -NH in 6.05 ppm were disappeared. Moreover, the integration of 8.3–6.4 ppm was decreased by 1, which proved that the -NH was in 8.3–6.4 ppm and the successful synthesis of FPD. In Figure 1c, the peak in ^31^P NMR spectrum showing at 28.9 and 31.2 ppm revealed that DOPO was successfully attached on the monomer. FT-IR was also taken for further structure identification. As can be seen in Figure 1d, the absorption peaks at 3370 and 3068 cm^−1^ were assigned to the N-H bond and Ph-H bond, and a series of peaks located at 1198 (P-O-C stretching) and absorption peaks at 1276 (P=O bond) and 1610 (P-Ph) cm^−1^ stood for DOPO [33]. All these evidences proved that the FPD as the target product was successfully synthesized. 

In the previous research, mussel-inspired improvement of the interfacial performance of fiber-reinforced composites was always a co-deposition of catechol onto fiber and subsequently prepared modified fiber reinforced composites [24]. The reported chemical compound DPDDM [14], containing the catechol structure agent, was utilized as a co-curing agent for epoxy resin and it was added to the epoxy resin directly. What is more, we found that it was insolube in any organic solvent except DMF due to the strong rigidity of the structure, which indicates that it cannot achieve the adhesion function of catechol and be used for the modification of composite materials. In this way, the FPD was designed to overcome the solubleness problem. To further explore its solubleness, FPD was placed in a common low-boiling-point solvent, such as alcohol, acetone, dichloromethane, trichloromethane, and tetrahydrofuran, and the results showed that the FPD could dissolve in tetrahydrofuran with a high solubility and the image of dissolution status was demonstrated in Figure S3. 

**Figure 2:**
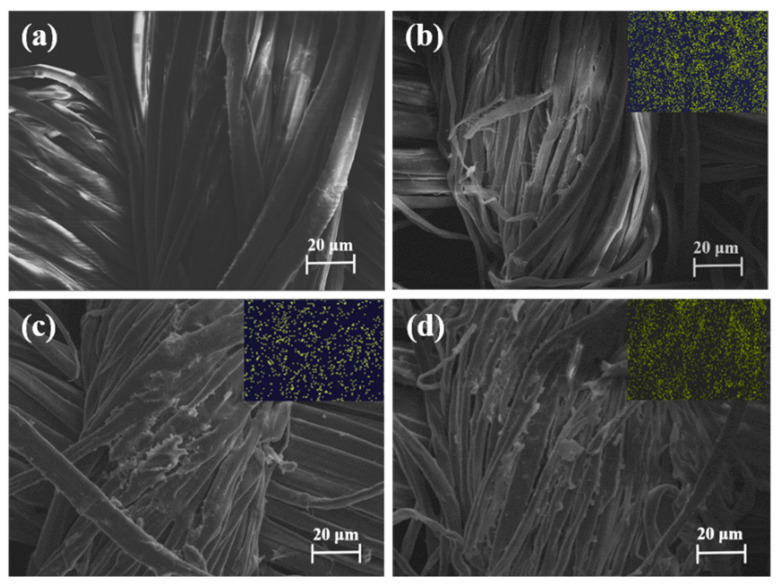
SEM micrographs and their corresponding elemental mappings of P element for RF-0 (**a**), RF-1 (**b**), RF-2 (**c**), and RF-3 (**d**).

To confirm whether the modification process was successful, SEM and EDS were characterized to analyze the uncoated (RF-0) and coated fabrics (RF-1, RF-2, and RF-3). Figure 2 presents the SEM pictures of the four kinds of fibers. The surface of the uncoated fabric was smooth and flat, as shown in Figure 1a. The SEM images in Figure 2b–d clearly show that FPD particles were deposited on the surface of ramie fibers. We further used EDS mapping to investigate the element distribution of FPD-treated ramie fabric. In summary, the above results confirm that FPD was successfully coated onto the ramie fiber. 

### 3.2. Mechanical Properties and Compatibility 

**Figure 3:**
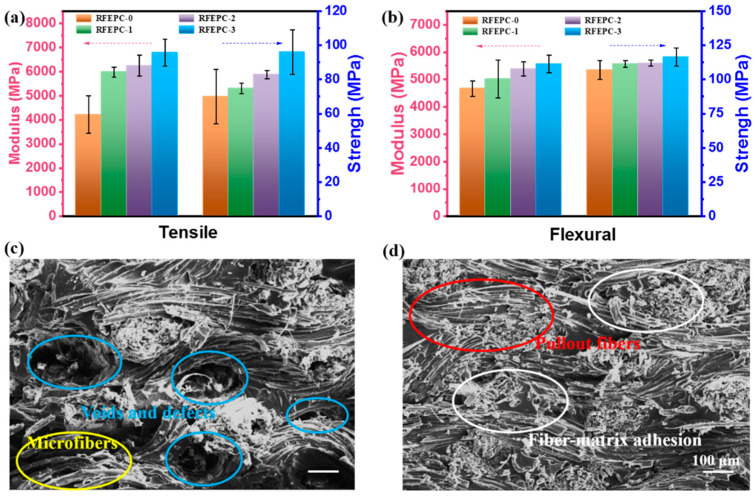
Mechanical properties for all composites: tensile strength and tensile modulus (**a**); flexural strength and flexural modulus (**b**); and SEM images of fracture surfaces of composites after tensile test: RFEPC-0 (**c**) and RFEPC-3 (**d**).

In this work, FPD acted with a role of linking the matrix and reinforcement of the composites, and it was expected that the FPD would confer the composites’ better mechanical properties and interface performance. By comparing the mechanical behavior of composites in Figure 3 and Table 2, as can be seen, the flexural strength, flexural modulus, tensile strength, and modulus of RFEPC-0, RFEPC-1, RFEPC-2, and RFEPC-3 increased in that order. Namely, the maximum flexural modulus and tensile modulus of 5570 MPa and 6779 MPa were observed for RFEPC-3, which was 19.3% and 60.9% higher than that of RFEPC-0 (4670 MPa and 4212 MPa), respectively. With regard to the flexural and tensile strength, it an enhancing trend from 107 MPa and 70 MPa for RFEPC-0 to 116.5 MPa and 96 MPa for RFEPC-3, respectively, was also demonstrated. The impact strength was also increased from 5.61 for RFEPC-0 to 7.22 kJ·m^−2^ for RFEPC-3. Hence, it can be concluded that the presence of the FPD did enhance the mechanical performance. Moreover, it is well known that the mechanical properties of fiber-reinforced composites are greatly influenced by the interfacial bond strength.

To further acquaint the improvement of mechanical composites, the fractured surfaces of the composites after the tensile test were observed by SEM micrographs. As shown in Figure 3c, it was easy to identify the ramie fibers with long and smooth surfaces were pulled out in clusters from the matrix, leaving some voids [34]. It can be observed that most of the fractured surfaces were fiber (indicated by yellow dotted circles) rather than epoxy resin, and it was hard to identify the interface between ramie fiber and resin, and some obvious voids (indicated by blue dotted circles) could be identified easily. This morphology basically indicated that there was little or no epoxy resin adhering on the surface of the pulled-out-ramie fabric, namely a very weak interfacial adhesion between fibers and the matrix. From Figure 3d, the ramie fibers seem to be trapped within the resin matrix since no obvious pulled-out fibers and voids were observed (indicated by red and white dotted circles), suggesting a much stronger interfacial interaction between the FPD-treated ramie fabric and the resin matrix, indicating that compatibility was strengthened, and thus the stress from matrix to reinforcements can be transferred efficiently, avoiding stress concentrations, which leads to higher tensile and flexural strengths, as evidenced by the mechanical test.

**Figure 4:**
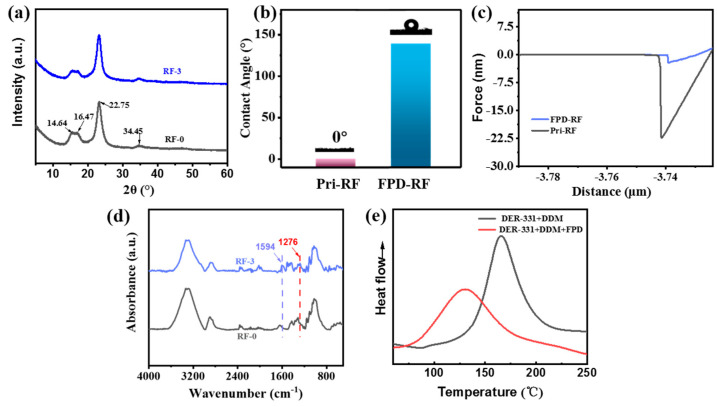
XRD spectra for RF-0 and RF-3 (**a**); water contact angle of RF-0 and RF-3 (**b**); adhesive force curves between RF-0, RF-3 and AFM tip (**c**); ATR-FTIR spectra for RF-0 and RF-3 (**d**); and DSC curves for FPD as the co-curing agent (**e**).

Normally, when the cellulose is treated by organic solvent, the cellulose on the surface of ramie fiber may be damaged to a certain extent; for example, the cellulose I in the crystalline region of ramie fiber gradually would change to the fiber II in the amorphous region, which leads to the mechanical properties of ramie fiber being decreased due to the decrease in crystallinity, and further lowering the mechanical properties of ramie fabric/epoxy resin composite [19,35]. The crystal structures of the three kinds of ramie fibers were analyzed by XRD, which is clearly shown in Figure 4a. The peak values of pristine ramie fiber were at 2θ = 14.64°, 16.47°, 22.75°, and 34.45° positions, corresponding to the crystal planes of cellulose (101), (110), (002), and (004), respectively [36,37,38]. The crystal structure and the intensity of the peak of the modified ramie fiber were basically consistent with that of the original one. These structures indicate that both the two chemical modifications never changed the crystal structure of ramie fiber, which means that the mechanical property of the ramie fiber was not damaged.

The poor compatibility between the polymer matrix and untreated ramie fiber is a very significant factor for the weak mechanical performance. The compatibility between the polymer matrix and ramie fiber would be facilitated via reducing the polarity difference between ramie fiber and epoxy resin. In this paper, the compatibility between the two phases was increased by improving the hydrophobicity of ramie fiber. To verify whether the FPD could change the waterproof property of ramie fiber, the water contact angle and AFM were tested. It was a surprise to find that the water contact angle of the ramie fabric modified by FPD could reach 138.7° (Figure 4b); this may due to the fact that FPD was intrinsically structurally rigid and hydrophobic. Moreover, the adhesive forces between a standard silicon nitride AFM tip and fibers were quantitatively characterized by AFM of the PF-QNM mode. A clear adhesion force–distance curve among the different samples can be observed on Figure 4c. It was interesting to observe that there was a big gap for the mean adhesion force between pristine fiber and FPD-modified fabric, which was because the AFM tip was hydrophilic, leading the adhesion forces between the hydrophilic AFM tip and the hydrophobic FPD-modified fiber to decrease, which was highly echoed with the water contact angle result. The enhanced hydrophobicity of ramie might be attributed to the FPD that was hydrophobic and it was successfully incorporated into the ramie fabric. In addition to the SEM analysis, ATR-FTIR was measured as well, and the result was shown in Figure 4d, the broad peak between 3000 and 3600 cm^−1^ was attributed to the O-H stretching vibration of the cellulose. Similarly, the peaks observed in the region of 800 to 1200 cm^−1^ were also attributed to cellulose [39]. Comparing the spectrum of the pure fiber with that of FPD-treated ramie, the new characteristic peaks appearing at 1276 and 1594 cm^−1^ for the FPD-treated fabric attributed to the DOPO absorption could be observed clearly, which also demonstrates that the FPD was successfully coated onto the ramie fabric.

**Figure 5:**
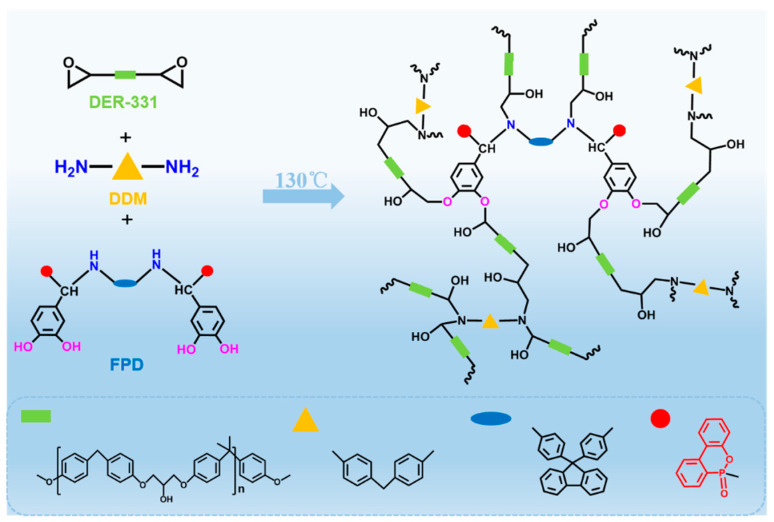
The possible reaction mechanism of FPD and 4, 4′-diaminodiphenylmethane (DDM) with DER-331.

In this work, FPD had a role of linking the matrix and reinforcement of the composites; in addition to the undamaged fiber and improved compatibility between fiber and matrix, FPD also could play the role of being a co-curing agent for the matrix, and to investigate whether the FPD could cure the epoxy resin, DSC was tested and the results are shown in Figure 4e. Interestingly, there is only one exothermic peak when the PFD acts as the co-curing agent for epoxy resin. Additionally, the temperature of the opening ring was also decreased, which avoids the mechanical destroying of plant fiber when exposure high temperature for long time. What is more, the possible reaction mechanism of FPD and 4, 4′-diaminodiphenylmethane (DDM) with DER-331 is illustrated in Figure 5. 

### 3.3. Thermal Stability and Flame Resistance Performance of Composites

**Figure 6:**
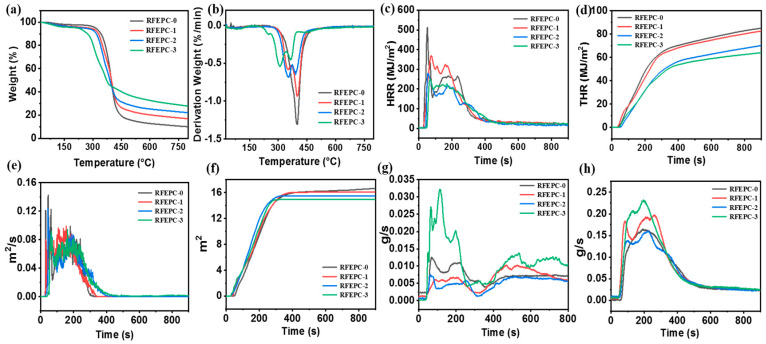
TGA (**a**) and DTG curves (**b**), HRR (**c**), THR (**d**), SPR (**e**), TSP (**f**), COP (**g**), and CO_2_P (**h**) and curves for composites.

The thermal degradation and stability of the material have important effects on the anti-flame performance. Hence, TGA was assessed to evaluate the thermal decomposition process under nitrogen atmosphere of all samples. As displayed in Figure 6 and Table 3, the thermal stability of the samples obviously showed an improvement with the addition of FPD. Accordingly, as shown in Figure 4a,b, the T_d10_ (the temperature of 10% mass loss) of the three cured composites were tiny and declined in the order of RFEPC-0, RFEPC-1, RFEPC-2, and RFEPC-3 (342, 329.3, and 318 vs. 272 °C), indicating that FPD led to the earlier decomposition. This was ascribed to FPD and began to decompose first at a lower temperature to form phosphoric acid or polyphosphate compound since the lower thermal stability of P-C and O=P-O bonds in FPD, compared with the C-C bond [40]. In addition, the carbon residue at 800 °C was increased in that order, the highest char residues value of 27.77% was obtained for RFEPC-3, which was higher than that of RFEPC-0, only 9.92%, and might indicate the improved thermal stability flame safety of composites after the FPD incorporation.

The flammability of the composites was investigated by the limit oxygen index (LOI). Generally speaking, the value of the LOI of RFEPC-0 was only 23.4%. What is more, the LOI value increased to 34.6% rapidly after 30% FPD was modified, which demonstrated that the flame retardancy increased after adding FPD. 

**Figure 7:**
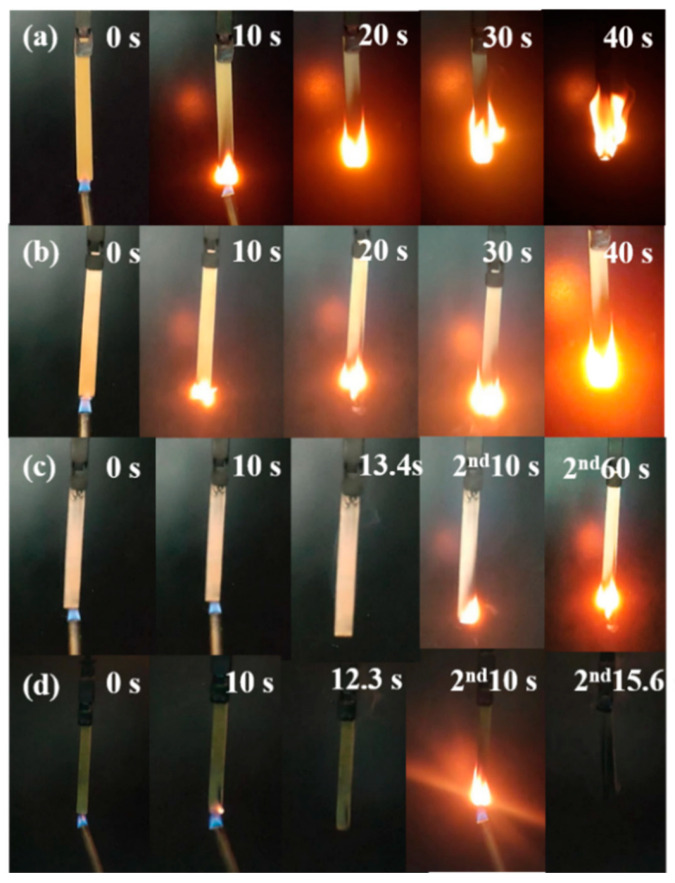
Digital photographs of RFEPC-0 (**a**), RFEPC-1 (**b**), RFEPC-2 (**c**), and RFEPC-3 (**d**) during the vertical test.

Further assessment of the flammability of the ramie/epoxy resin composites with different loading of FPD was conducted by vertical burning test. The obtained data and images are collected in Table 3 and Figure 7. Since ramie and epoxy resin are flammable materials, RFEPC-0 was completely consumed during the UL-94 tests, and no rating was obtained. Things changed better in the presence of 20% FPD, but it was still inhibited to some extent, and the flame retardancy was not enough to extinguish fire itself. It was noticed that RFEPC-3 self-extinguished within 10 s after the second fire was removed, rarely producing smoke during the combustion process, which indicates that FPD imparts to the composite high-flame retardancy property. 

The cone calorimeter test is normally analyzed to evaluate the combustion performance of material in actual fire condition, and the heat release rate (HRR) and total heat release (THR) are two important indicators; the higher HRR or THR, the greater risk of fire for the material. The cone calorimeter test results for composites were exhibited in Figure 6. Both HRR and THR were significantly decreased with the increasing loading of FPD. Accordingly, the HRR and THR declined from 513.8 W/m^2^ and 85.13 W/m^2^ for RFEPC-0 to 258.9 kW/m^2^ and 64.23 kW/m^2^ for RFEPC-3, respectively. This trend was similar to that observed for the flammability tests, and in agreement with the TGA results. In addition, Figure 6e,f reveals that FPD could slow down the smoke production rate (SPR) and the total smoke production (TSP) of composites, illustrating that FPD could efficiently suppress the smoke formation during burning, and thus improving the fire safety of composites. The smoke suppression might be relevant with the excellent char-forming efficient of FPD, which not only isolates and protects the materials inside from fire, but also restrains the diffusion of the produced flammable gases [41]. Interestingly, the CO production rate (COP) for the RFEPC-3 increased, which may be attributed to the fact that the PO· and PO_2_· free radicals released from DOPO can be effectively quenched and eliminated from the free radical chain reaction, resulting in incomplete combustion of the matrix. Moreover, the CO_2_ production rate (CO_2_P) for the RFEPC-3 also increased, which could take away the heat as well as dilute the flammable gas and oxygen in the gas phase and indicates the presence of the gaseous-phase flame-retardant effect of FPD [42]. 

To sum up, through the analysis of thermal stability, LOI, UL-94, and CCT behavior, FPD-treated ramie fiber/epoxy resin composite exhibited obvious flame resistance and RFEPC-3 performed the best fireproof manifestation.

Based on above results, it was concluded that the addition of FPD could not only significantly improve the thermal and flame retardancy of ramie fiber/epoxy resin composites, but also simultaneously increase its mechanical properties, including flexural, tensile, and impact. Compared with the previous work [35], ammonium polyphosphate (APP) was used as a flame-retardant modifier and the composite with 8.9% flame retardant could achieve a UL-94 V-0 rating. However, slight damage in flexural strength or modulus under different APP loading appeared with the composite without APP as the control, which indicated that FPD was more of an all-round modifier than APP. 

### 3.4. Flame-Retardant Mechanism

#### 3.4.1. The Analysis of Thermal Degradation Gaseous Products

**Figure 8:**
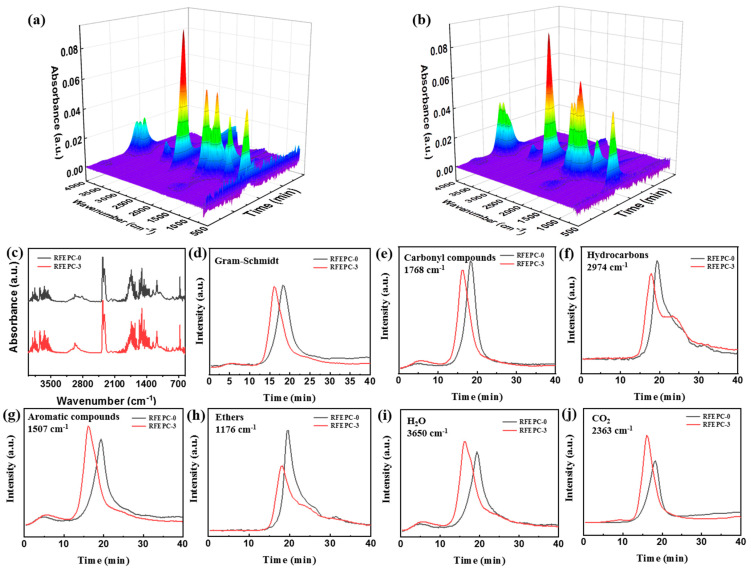
TG-FTIR 3D spectra for RFEPC-0 (**a**) and RFEPC-3 (**b**); FT-IR spectra of pyrolysis products for RFEPC-0 and RFEPC-3 at maximum decomposition rate (**c**); intensity of pyrolysis products (total (**d**), carbonyl compounds (**e**), hydrocarbons (**f**), aromatic compounds (**g**), ethers (**h**), H_2_O (**i**), and CO_2_ (**j**) versus time for RFEPC-0 and RFEPC-3.

TG-FTIR was performed in order to analyze gaseous products of materials during the thermal decomposition process and further unveil the gaseous-phase action of flame retardants, as illustrated in Figure 8. As depicted in Figure 8a,b, the absorption positions of FTIR peaks in 3D images were quite similar, but the absorption intensities of RFEPC-0 were stronger than that of RFEPC-3, which implied that the introduction of flame retardants would suppress the thermal degradation process rather than change it. As displayed in Figure 8c, with RFEPC-3, the P-O absorption peak appeared at 1060 cm^−1^, indicating that the phosphorus in FPD played a fire-resistant role in the gaseous phase. Figure 8d was the absorption peak intensity of the main thermal degradation products that changed with time, the time of gas-phase degradation products of RFEPC-3 was earlier, and the gas release rate was lower, which mainly indicated the catalytic degradation effect of flame retardants on the composites, consistent with the results of TGA results. The release of carbonyl compounds was declined for RFEPC-3 when compared with RFEPC-0 (Figure 8e), which indicates that the free radical cracking reaction was effectively inhibited in the RFEPC-3 system. Meanwhile, the release of flammable gases such as hydrocarbons (Figure 8f), aromatic compounds (Figure 8g), and ethers (Figure 8h) was also restrained, while the production of nonflammable gases such as water (Figure 8i) and carbon dioxide (Figure 8j), can be promoted, which could take away the heat as well as dilute the flammable gas and oxygen in the gas phase. In a word, FPD could improve the flame retardancy through facilitating the release of non-flammable gases such as H_2_O and CO_2_, and restraining the release of flammable gases including hydrocarbons, carbonyl, aromatic, and ethers compounds, etc. These CO_2_ trend results are consistent with the CO_2_P value in the CCT test.

#### 3.4.2. The Characteristic of the Condensed Phase

**Figure 9:**
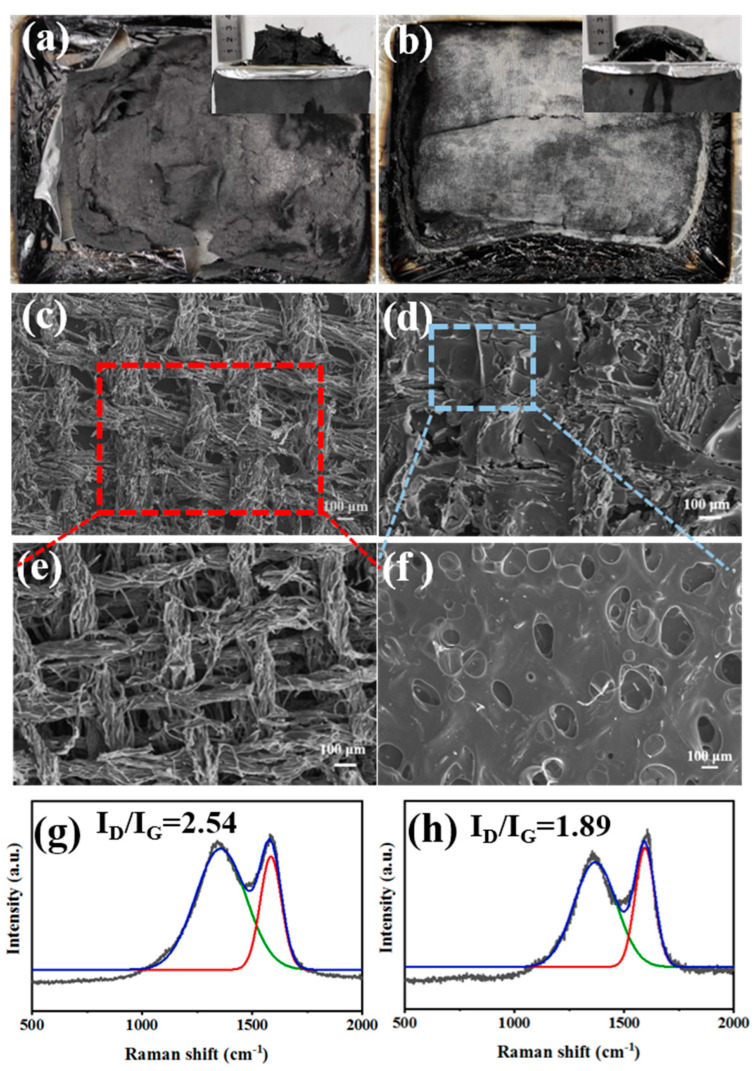
The vertical and the horizontal views of the char residue of RFEPC-0 (**a**) and RFEPC-3 (**b**) after the cone calorimeter test; SEM micrographs of char residue of RFEPC-0 (**c**,**e**), and RFEPC-3 (**d**,**f**) under different magnificent; and Raman spectra of residual chars for RFEPC-0 (**g**) and RFEPC-3 (**h**).

**Figure 10:**
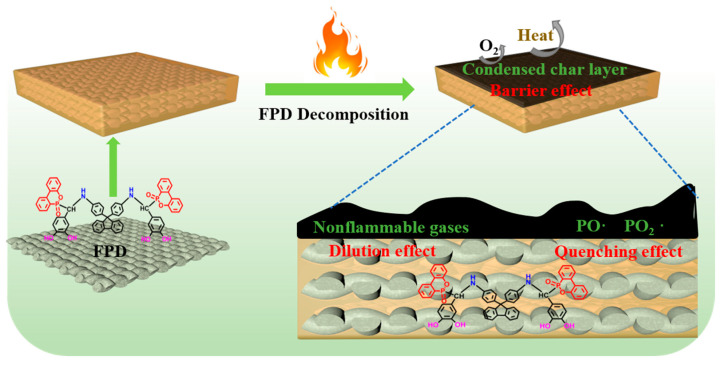
The schematic diagram of the possible flame-retardant mechanism of ramie fiber/epoxy composites modified by FPD.

Normally, besides releasing non-flammable gases in the gaseous phase, the DOPO-containing flame retardants also can exert flame-retardant effects through promoting the formation of a dense char layer in the condensed phase. To further ascertain the flame-retardant mechanism, the condensed phases obtained after cone calorimeter tests for RFEPC-0 and RFEPC-3 were investigated. As shown in Figure 9a, some char residue was observed for RFEPC-0, and the char residue was broken and discontinuous. This was probably ascribed to the rapid decomposition of composites, which would break through the thin char layer. On the contrast, that the char layer became more compact, continuous, and intumescent could be observed since the addition of FPD leading the char residue increased and gentler degradation happened, as displayed in Figure 9b. Further analyzing the morphology of char residue by SEM (Figure 9c,e), it was found that the char residue of RFEPC-0 exhibited the homogeneous and complete morphology of burned fiber structures, which may be attributed to the incomplete combustion of composites. Conversely, the incorporation of FPD, which is demonstrated in Figure 9d and f, made the char residue be denser and only a few fibers could be observed; the fiber would be protected by epoxy resin, which also can indicate that the FPD did participate in the curing process of epoxy resin. Furthermore, the combination of a compact external char layer and hollow char fiber residue was capable of storing considerable pyrolysis gases with masses of phosphorous free radicals and nonflammable gases during combustion, which were instantly released to snuff out the flame when the gas pressure in the interior was sufficient to break through the char layer. The Raman spectra also demonstrated the former conclusion. As we know, the ratio of two peak areas (ID/IG) represents the graphitization degree, which is the ratio of disordered graphite (located at 1350 cm^−1^) and ordered graphite (located at 1590 cm^−1^), which means the lower ID/IG is, the more regular the char layer is [15]. Therefore, compared with RFEPC-0 (Figure 9g) and RFEPC-3 (Figure 9h), RFEPC-3 exhibited a lower ID/IG than that of RFEPC-0. Based on the above results, a supposed mechanism is presented for the enhanced flame retardancy. In a word, the integrated and continuous char layer was more conducive to prevent the heat transfer, O_2_, and flammable volatiles, revealing a strong barrier effect to improve the fire safety of the epoxy composites. Based on the above analysis, the schematic diagram of the possible flame retardant mechanism of ramie fiber/epoxy composites modified by FPD was demonstrated in Figure 10. 

## 4. Conclusions

In this study, inspired by mussel, a multi-functional modifier, which could improve the mechanical and thermal performance of ramie-reinforced epoxy resin composites, simultaneously was designed and synthesized successfully through just one step. It can be successfully introduced and firmly adhere to the ramie fabric, and the hydrophobic property and N-H were both integrated into ramie fabrics so the compatibility between fiber and epoxy resin could be facilitated; and thus, FPD can play a bridging role to enhance the interfacial properties of ramie epoxy composites. The flexural and tensile strength and modulus were all increased, from 107 MPa, 70 MPa, 4670 MPa, and 4212 MPa for RFEPC-0 to 116.5 MPa, 96 MPa, 5570 MPa, and 6779 MPa for RFEPC-3. In addition, the fire safety of modified RFEPC was also improved by forming compact and continuous char layers and facilitating the release of non-flammable gases, which enabled RFEPC-3 to achieve a UL-94 V-0 rating. This work offers an effective approach to fabricate strong interfacial and flame-retardant ramie performance-reinforced epoxy composites, which may enable potential application prospects in the files of construction, trains, and automobiles with interior trim. 

## Figures and Tables

**Table 1:** Formulations of different samples.

Samples	Ramie Fiber (g)	DER-331 (g)	DDM (g)	FPD (g)	Content of P (wt.%)
RFEPC-0	120	120	30.3	0	0
RFEPC-1	120	120	29.6	12	1.70
RFEPC-2	120	120	28.9	24	3.38
RFEPC-3	120	120	28.2	36	5.04

**Table 2:** The mechanical properties of different samples.

Sample	Tensile		Flexural		Impact Strength (kJ·m^−2^)
	Strength (MPa)	Modulus (MPa)	Strength (MPa)	Modulus (MPa)	
RFEPC-0	70 ± 16	4212 ± 776	107 ± 7	4670 ± 276	5.61 ± 0.27
RFEPC-1	74.8 ± 3	5979 ± 207	111 ± 2	5020 ± 688	6.17 ± 0.21
RFEPC-2	83 ± 2.4	6248 ± 435	112 ± 2.2	5390 ± 252	6.83 ± 0.15
RFEPC-3	96 ± 13	6779 ± 563	116 ± 6. 7	5570 ± 322	7.22 ± 0.24

**Table 3:** Results of thermal stability and flame retardant measurements.

Samples	T_d10_ (°C)	Char Yield at 800 °C (%)	Rmax (%/°C)	LOI (%)	t_1_/t_2_ (s)	Dripping	UL-94
RFEPC-0	342.3	9.92	398.7	23.4	- ^a^	N	No-rating
RFEPC-1	329.3	16.84	399	26.8	- ^a^	N	No-rating
RFEPC-2	318.3	22.05	353	29.6	- ^a^	N	No-rating
RFEPC-3	272.3	27.77	309	34.6	2.3/5.6	N	V-0

- ^a^: Does not extinguish fire after ignition.

## Data Availability

Not available.

## Supplementary Materials

The following are available online at https://www.mdpi.com/article/10.3390/polym15183800/s1, Figure S1: ^13^C NMR spectra of FPD, Figure S2: ^1^H NMR spectra with D_2_O of FPD, Figure S3: The image of FPD dissolution status in alcohol (a), acetone (b), dichloromethane (c), trichloromethane (d) and tetrahydrofuran (e).
