# Supplementary material for: Preparation and Application of a Multifunctional Interfacial Modifier for Ramie Fiber/Epoxy Resin Composites
Source: Polymers (Basel). 2023 Sep 18;15(18):3800. doi: 10.3390/polym15183800 (PMC10537050; doi:10.3390/polym15183800)
Supplement: Supplementary file 1 [file polymers-15-03800-s001.zip › polymers-2587690-supplementary.pdf]

Preparation and Application of a Multifunctional Interfacial Modifier for Ramie Fiber/Epoxy Resin Composites

Liyue Zhang [1,2], Jingkai Liu [2], Jinyue Dai [2], Xufeng Zhang [3], Xiaoling Liu [1], Xiaoqing Liu [2,*] and Xiaosu Yi [1,*]

[1] New Material Institute, University of Nottingham Ningbo China, Ningbo 315100, China; zhangliyue@nimte.ac.cn (L.Z.); xiaoling.liu@nottingham.edu.cn (X.L.)
[2] Key Laboratory of Marine Materials and Related Technologies, Ningbo Institute of Materials Technology and Engineering, Chinese Academy of Sciences, Ningbo 315201, China; liujingkai@nimte.ac.cn (J.L.); daijinyue@nimte.ac.cn (J.D.)
[3] College of Materials, Beijing Institute of Technology, Beijing 100081, China; 010xufeng@sina.com
* Correspondence: liuxq@nimte.ac.cn (X.L.); xiaosu.yi@nottingham.edu.cn (X.Y.)

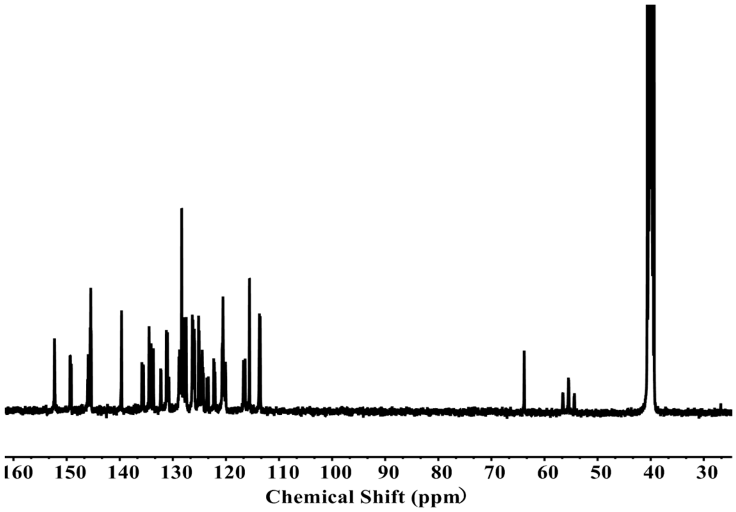

Figure S1 ^{13}C NMR spectra of FPD.

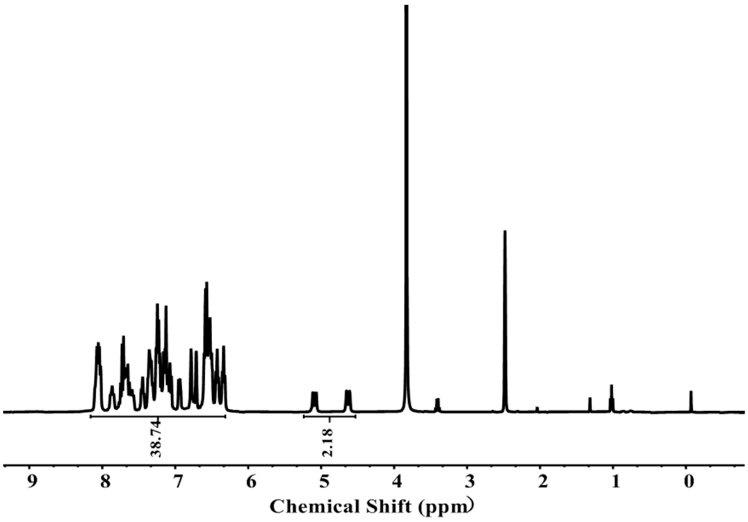

Figure S2 ^{1}H NMR spectra with D_2O of FPD.

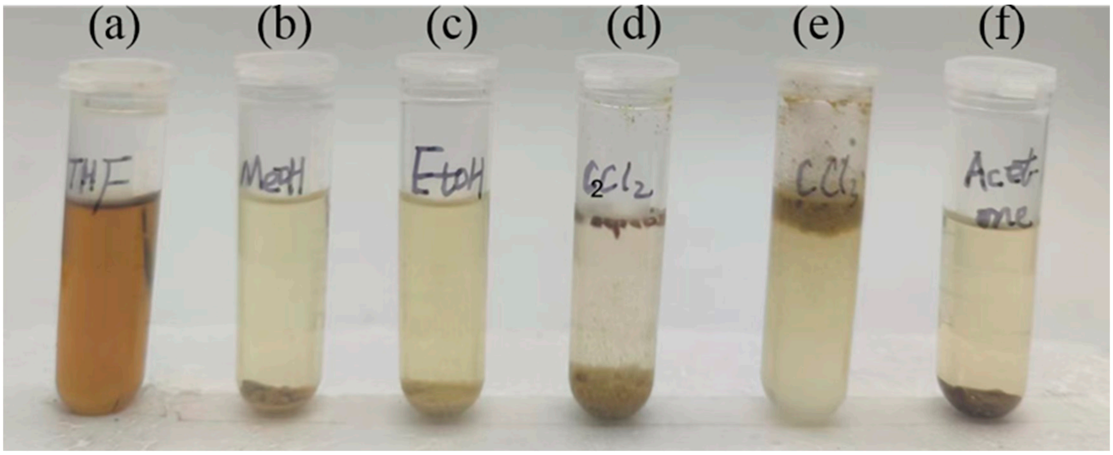

Figure S3 The image of FPD dissolution status in tetrahydrofuran(a), methyl alcohol(b), ethyl alcohol(c), dichloromethane(d), trichloromethane(e) and acetone(f).
